# “Adipose-derived mesenchymal stem cell therapy for the management of female sexual dysfunction: Literature reviews and study design of a clinical trial”

**DOI:** 10.3389/fcell.2022.956274

**Published:** 2022-09-28

**Authors:** Van T. Hoang, Hoang-Phuong Nguyen, Viet Nhan Nguyen, Duc M. Hoang, Tan-Sinh Thi Nguyen, Liem Nguyen Thanh

**Affiliations:** ^1^ Vinmec Research Institute of Stem Cell and Gene Technology, Vinmec Health Care System, Hanoi, Vietnam; ^2^ Vinmec International Hospital—Times City, Vinmec Health Care System, Hanoi, Vietnam; ^3^ College of Health Science, Vin University, Vinhomes Ocean Park, Hanoi, Vietnam

**Keywords:** sexual function impairment, female sexual dysfunction, adipose-derived mesenchymal stem cells, cell therapy, regenerative medicine, menopause, ASCs (adipose stem cells)

## Abstract

Hormone imbalance and female sexual dysfunction immensely affect perimenopausal female health and quality of life. Hormone therapy can improve female hormone deficiency, but long-term use increases the risk of cardiovascular diseases and cancer. Therefore, it is necessary to develop a novel effective treatment to achieve long-term improvement in female general and sexual health. This study reviewed factors affecting syndromes of female sexual dysfunction and its current therapy options. Next, the authors introduced research data on mesenchymal stromal cell/mesenchymal stem cell (MSC) therapy to treat female reproductive diseases, including Asherman’s syndrome, premature ovarian failure/primary ovarian insufficiency, and vaginal atrophy. Among adult tissue-derived MSCs, adipose tissue-derived stem cells (ASCs) have emerged as the most potent therapeutic cell therapy due to their abundant presence in the stromal vascular fraction of fat, high proliferation capacity, superior immunomodulation, and strong secretion profile of regenerative factors. Potential mechanisms and side effects of ASCs for the treatment of female sexual dysfunction will be discussed. Our phase I clinical trial has demonstrated the safety of autologous ASC therapy for women and men with sexual hormone deficiency. We designed the first randomized controlled crossover phase II trial to investigate the safety and efficacy of autologous ASCs to treat female sexual dysfunction in perimenopausal women. Here, we introduce the rationale, trial design, and methodology of this clinical study. Because aging and metabolic diseases negatively impact the bioactivity of adult-derived MSCs, this study will use ASCs cultured in physiological oxygen tension (5%) to cope with these challenges. A total of 130 perimenopausal women with sexual dysfunction will receive two intravenous infusions of autologous ASCs in a crossover design. The aims of the proposed study are to evaluate 1) the safety of cell infusion based on the frequency and severity of adverse events/serious adverse events during infusion and follow-up and 2) improvements in female sexual function assessed by the Female Sexual Function Index (FSFI), the Utian Quality of Life Scale (UQOL), and the levels of follicle-stimulating hormone (FSH) and estradiol. In addition, cellular aging biomarkers, including plasminogen activator inhibitor-1 (PAI-1), p16 and p21 expression in T cells and the inflammatory cytokine profile, will also be characterized. Overall, this study will provide essential insights into the effects and potential mechanisms of ASC therapy for perimenopausal women with sexual dysfunction. It also suggests direction and design strategies for future research.

## Introduction

Adipose tissue is an important production site of female sex hormones outside the ovaries (Ahima and Flier 2000; Hetemäki et al., 2021). In premenstrual women, adipocytes express a high level of estrone and metabolic enzymes to convert this form into estrogen (Si et al., 2019). Adipose tissue is also an abundant source of mesenchymal stem/stromal cells (MSCs). These multipotent cells reside in the stromal vascular fraction of adipose tissue and represent a component of the vascular niche with an essential role in the regulation of angiogenesis as well as tissue repair upon injury (Lin et al., 2008; Panina et al., 2018). Adipose-derived stromal/stem cells (ASCs) are known as key players in adipogenesis (Matsushita and Dzau 2017; Munir et al., 2017; Q. Chen et al., 2016). Both MSC-based therapy and drugs controlling MSC maturation into white and brown fat have been studied to manage obesity (Jaber et al., 2021).

In regenerative medicine, ASCs represent a robust and potent therapeutic candidate that can be used directly after isolation or expanded on a large scale *in vitro* (Bateman et al., 2018; Kabat et al., 2020). Both autologous and allogeneic ASCs have been licensed for the treatment of several diseases, such as Alofisel to treat complex perianal fistulas in Crohn’s disease in the EU, Allosterm for bone regeneration in the United States, and QueenCell, Cupistem, and Adipocel to treat subcutaneous tissue defects and Crohn’s fistula in Korea (Najar et al., 2022). Furthermore, ASC therapy has been applied to treat other disorders, such as bone and cartilage degenerative diseases, ischemic disorders, cardiovascular diseases, neurological disorders, autoimmune diseases, wound healing, and skin burns (Si et al., 2019; Krawczenko and Klimczak 2022). In the case of female reproductive diseases, most experimental studies and clinical trials have used ASCs for patients with premature ovarian failure and Asherman syndrome (Zhao et al., 2019; Na and Kim 2020). ASCs were able to improve sex hormone levels and restore fertility in patients (Polonio et al., 2021). Studies in animal models also support clinical results demonstrating ASCs as a promising novel therapeutic opportunity for female infertility (L. Chen et al., 2018). This study reviews the mechanisms and outcomes of MSC therapy for female reproductive disorders. In addition, the authors introduce the study protocol of a to be initiated clinical trial: “evaluation of autologous ASC efficacy for the treatment of female sexual dysfunction: a randomized phase II crossover study”.

### Female sexual dysfunction as a widespread distress

**FIGURE 1:**
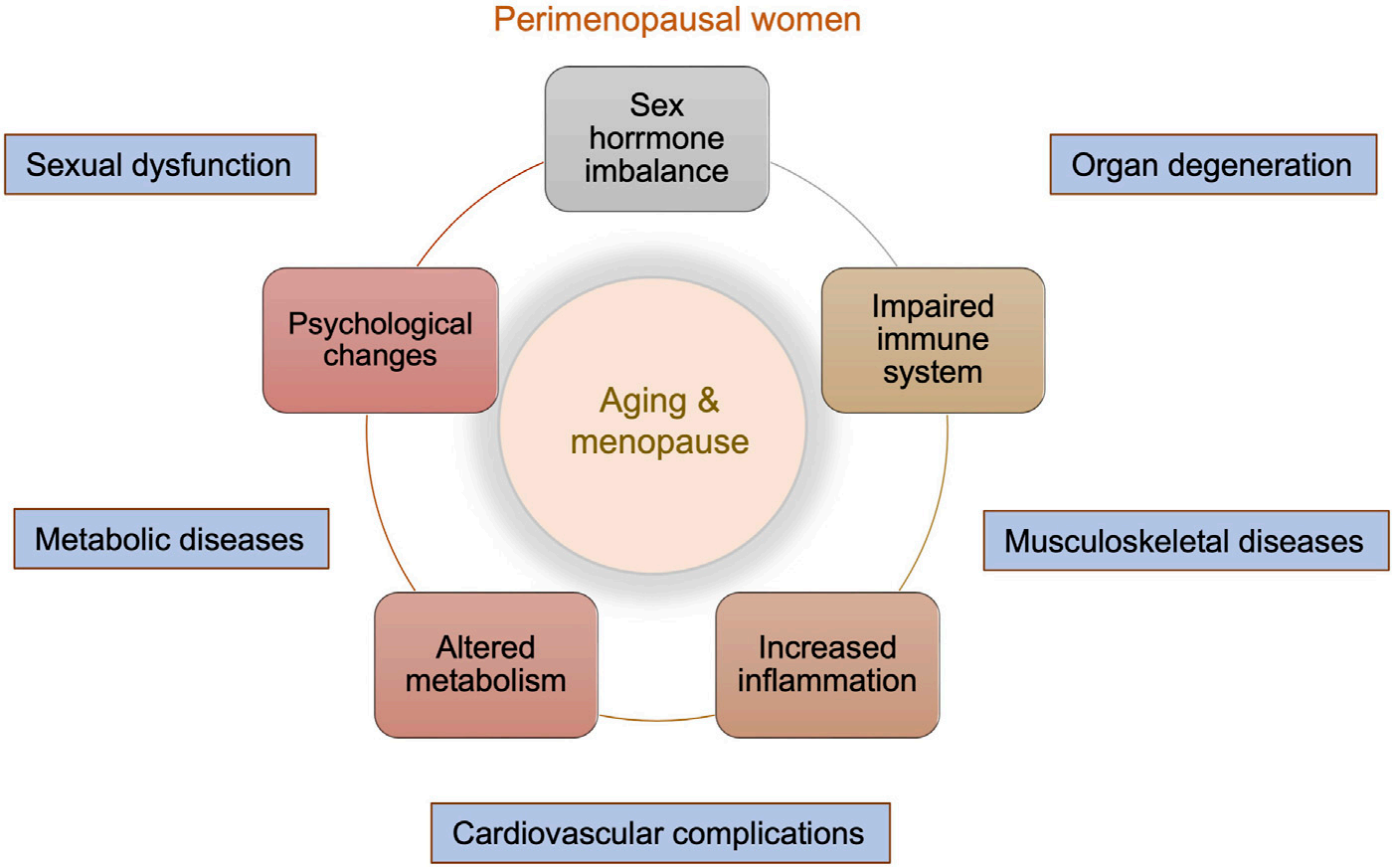
Overview of physical and emotional changes in perimenopausal women. Menopause is a natural aging process and marks enormous changes in both the general and sexual life of women. These include sex hormone imbalance, impaired immune system, increased inflammation, altered metabolism, and psychological changes. These changes can lead to accelerated organ degeneration and increased risks of musculoskeletal diseases, cardiovascular complications, metabolic diseases, and sexual dysfunction.

Female sexual dysfunction is highly prevalent–although it is not limited to–in aged women and widely impacts the health and quality of life of patients (Anastasiadis et al., 2002). The most common symptoms of female sexual dysfunction include diminished vaginal lubrication, pain during intercourse, lack of desire for sex, and difficulty in achieving orgasm (“American College of Obstetricians and Gynecologists' Committee on Practice Bulletins—Gynecology, 2019; Krakowsky and Grober 2018; Allahdadi et al., 2009). A survey in the United States showed that 43.1% of women reported having sexual problems, and 22.2% were diagnosed with sexually related distress based on the Female Sexual Distress Scale (FSDS) (Shifren et al., 2008). In four European countries, including France, Italy, Germany, and the UK, low sexual desire was the most frequent female sexual dysfunction, ranging from 21 to 36% depending on the country studied, and the prevalence was closely correlated with increasing age (Graziottin 2007). Between 70 and 80% of Finnish women aged 55 to 74 had decreased libido compared to 20% of those younger than 25 years (McCabe et al., 2016). Of note, menopause occurs in Caucasian and Asian women on average at age 51 (Boulet et al., 1994; Baber et al., 2016), and the deceased level of estrogens is associated with diverse physiological and emotional changes in postmenopausal women (Graziottin and Leiblum 2005). Along with the natural aging process, which is hallmarked by altered immune system functions, increased inflammation, and altered metabolism, the lack of sex hormones can further negatively impact women’s general and sexual health (Figure 1).

Many factors might influence female sexual dysfunction, including the endocrine system, medication side effects, overall health, and psychological and social life (Basson et al., 2001; Graziottin et al., 2006; Giraldi and Wåhlin-Jacobsen 2016). Age-related physical changes, such as a decrease in sex hormones, can directly cause sexual dysfunction (Davis and Tran 2001; R. Basson 2007; AlAwlaqi et al., 2017; McCoy and Davidson 1985). Several studies have demonstrated that an insufficient level of estrogen rather than testosterone is responsible for increased sexual distress during menopause (Dennerstein et al., 2002; Aziz et al., 2005; Gerber et al., 2005). Estrogen, especially estradiol, plays an important role in the development and regulation of female reproductive organs as well as the cardiovascular and cognitive system, bone integrity, and insulin sensitivity (Hall et al., 2001; Belgorosky et al., 2009; Findlay et al., 2010; Simpson and Santen 2015). Circulating estrogen is produced mainly by the ovaries and by the adrenal glands and placenta in smaller amounts in reproductive females (Hillier et al., 1994; J. Cui, Shen, and Li 2013). The estradiol level in serum drops after menopause, resulting in vaginal dryness and atrophy, reduced genital sensitivity upon stimuli and therefore lower sexual desire of middle-aged and older women (Bygdeman and Swahn 1996; Dennerstein et al., 2002; R. Basson 2007). Furthermore, a low level of estrogen will lead to reduced physiological needs, menstrual disorders, and difficulties in conceiving and nurturing the fetus (Nelson 2008; Gracia and Freeman 2018; Minkin 2019). Parallel to the change in estrogen, a gradual decline in testosterone belongs to a natural aging process of the body (Graziottin and Leiblum 2005). Testosterone is critical for sexual desire as well as sense of well-being in females (Davis and Tran 2001). Indeed, women with testosterone deficiency, most frequently observed in surgically menopausal patients, and those receiving antiandrogens reported low libido, reduced sexual interest and fatigue (Appelt and Strauss 1984; Adamopoulos et al., 1988; Bachmann 2002). In addition to decreased sex hormone levels, physical health conditions such as inflammatory, metabolic, and cardiovascular diseases also increase the risk of female sexual dysfunction (AlAwlaqi et al., 2017; Di Francesco et al., 2019; Lorenz 2019). A systematic review indicated that sexual dysfunction is common in patients with arthritis (Restoux et al., 2020). Patients with inflammatory bowel disease experience impaired sexuality depending on the disease severity (Timmer et al., 2008; de Silva et al., 2018; J. Zhang et al., 2022). Diabetes affects a wide range of patients’ health, including sexual activity. Many large studies have demonstrated an elevated sexual dysfunction prevalence among women with type 1 and type 2 diabetes (Enzlin et al., 2009; Nowosielski et al., 2010; Copeland et al., 2012; Shi et al., 2012; Elyasi et al., 2015). Hyperlipidemia, which is characterized by a pathogenic lipid profile and is known as a risk factor for vascular diseases, is linked to female sexual distress in all domains, including desire, arousal, lubrication, orgasm, satisfaction, and pain (Esposito et al., 2009; Martelli et al., 2012; Baldassarre et al., 2016). Data reporting a relationship between systemic arterial hypertension and sexual dysfunction in females remain controversial. While a significant correlation was observed in some studies (Doumas et al., 2006; Miner et al., 2012; De Franciscis et al., 2013; Nascimento et al., 2015), other studies reported no association between the diseases (Spatz et al., 2013; Foy et al., 2016). Overall, the data suggest that low serum sex hormone levels as well as disease-related conditions such as chronic inflammation, metabolic and cardiovascular diseases and persistent inflammation increase the risk of developing female sexual dysfunction.

Due to the complexity of the disorder, effective medical care for females with sexual dysfunction remains challenging. Estrogen replacement therapy is commonly prescribed to treat postmenopausal syndromes (Kovalevsky 2005; Simpson and Santen 2015; Stuenkel et al., 2015). Potential side effects of systemic and local use of estrogen include thrombosis, endometrial hyperplasia, stroke, and the development of breast and cervical cancer (Writing Group for the Women’s Health Initiative Investigators 2002; Wentzensen and Trabert 2015; Collaborative Group on Epidemiological Studies of Ovarian Cancer 2015). Moreover, the therapy negatively influences the serum testosterone concentration, leading to complications in sexual and general health, such as reduced libido and general sense of well-being, and/or increased muscle and bone loss (J. A. Simon 2002; Sarrel 2002). The use of androgen supplements for estradiol treatment was capable of reversing this effect (Davis et al., 1995; J. Simon et al., 1999; Shifren et al., 2000). Hence, combined therapy of estradiol and testosterone can be beneficial for the treatment of sexual dysfunction in menopausal women. However, long-term use of androgens might induce excessive hair growth in a male-like pattern (also known as hirsutism), acne, hair loss, decreased high-density lipoprotein levels, and hepatic toxicity (Abdallah and Simon 2007; Khera 2015). Flibanserin is the first FDA-approved drug to treat hypoactive sexual desire disorder, although there are still debates about its clinical benefit. The drug acts as a serotonin antagonist while enhancing the effect of dopamine to enhance the sexual response. In randomized trials, the treatment of women with hypoactive sexual disorder has shown modest efficacy compared to the control group (Jaspers et al., 2016; Lodise 2017). Adverse effects, including dizziness, sleepiness, nausea, and fatigue, were reported in these trials. Recently, the FDA approved bremelanotide for the treatment of hypoactive sexual desire disorder in premenopausal women (Mayer and Lynch 2020). The drug stimulates the neurological activity of the hypothalamic and limbic regions of the brain, probably via the dopamine signal to engage sexual activity (Pfaus et al., 2007). Although side effects appear mild to moderate with nausea, facial flushing, and headache, administration of the drug requires some effort, as it must be injected subcutaneously ca. 45 min prior to intercourse (Kingsberg et al., 2019). Despite the long history of searching for medication to treat female sexual dysfunction marked by the first use of estradiol in the 1940s, further research on novel therapy and the long-term safety of currently approved drugs remains essential, especially for those unsuitable for hormone replacement therapy.

### MSC therapy for the treatment of experimental female reproductive disorders

A potential benefit of cell therapy in the management of reproductive diseases was first reported for women whose ovaries were damaged after chemotherapy and whose ovarian function and fertility were recovered (Sanders et al., 1996; Hershlag and Schuster 2002). Recently, MSC therapy, including ASCs, has emerged as a potential candidate to regenerate damaged tissues and rejuvenate organs. Many studies have demonstrated the efficacy of MSCs from various sources in the treatment of diseases related to reproductive function and hormone deficiency. In a postmenopausal rat model, human umbilical cord-derived MSC infusion restored estradiol and AMH levels while decreasing FSH levels in correlation with increased levels of hepatocyte growth factor (HGF), vascular endothelial growth factor (VEGF) and insulin-like growth factor 1 (IGF-1), which play an important role in ovarian function (Jia Li B. et al., 2017). Human amniotic MSCs were able to restore AMH and estrogen levels in the ovaries of aging mice 1 week after stem cell injection (C. Ding et al., 2018a). In a rat model of Asherman’s syndrome, which is characterized by intrauterine adhesions due to the formation of scar tissue in the uterus, the administration of ASCs in combination with estrogen induced endometrial regeneration in treated animals (H. Sun et al., 2018).

An increasing number of studies have reported positive effects of MSC therapy on restoring ovarian function in premature ovarian failure (Zhao et al., 2019; Na and Kim 2020). Premature ovarian failure, also known as primary ovarian insufficiency, is defined as a loss of reproductive and hormonal functions of the ovaries in women before the age of 40 years. Premature ovarian failure results in a decline in women’s physical and mental health, such as amenorrhea, ovarian atrophy, sexual hypoactivity and infertility in young women (Beck-Peccoz and Persani 2006). In a rat model of chemotherapy-induced premature ovarian failure, BM-MSCs reduced luteinizing hormone (LH) and FSH and increased serum estradiol levels compared to the control group (Afifi and Reyad 2013). Placenta-derived MSCs restored serum estradiol, AMH, and FSH concentrations and recovered ovarian function in mice with premature ovarian failure (H. Zhang et al., 2018; Li et al., 2018). ASCs have also demonstrated promising potential to regenerate ovarian functions. Indeed, ASCs enhanced angiogenesis and recovered the number of follicles and corpus luteum defects in damaged ovaries (Terraciano et al., 2014; M. Sun et al., 2013). Improved serum estradiol levels and increased pregnancy rates have also been reported in ASC-transplanted mice (Fouad et al., 2016). Another approach was a combined injection of ASCs and collagen scaffolds. The treated group showed a significant increase in estradiol levels, granulosa cell proliferation, and both mating and pregnancy rates compared to the PBS control group (Su et al., 2016). A study in rabbits showed that MSC therapy improves ovarian function through direct differentiation into specialized cells in the ovary or secretion of VEGF growth factor, which helps ovary regeneration (Abd-Allah et al., 2013).

Recently, MSCs have been investigated for the treatment of vaginal atrophy. The disease affects more than half of menopausal women due to decreased estrogen levels resulting in thinning of vaginal epithelium and reduced local blood flow. As a result, women with vaginal atrophy often suffer from vaginal dryness, itching, burning, and pain when urinating and during intercourse (Naumova and Castelo-Branco 2018). ASCs and bone marrow-derived MSCs were able to improve epithelial thickness in a menopause rat model of vaginal atrophy with superior therapeutic effects in the former group (Kasap et al., 2019). The expression of estrogen receptor, VEGF and its receptor was increased in vaginal epithelium and connective tissue after MSC administration (Kasap et al., 2019). Zhang et al. performed *in situ* injection of umbilical cord-derived MSCs to repair fragile vaginal tissue in an ovariectomized rhesus macaque model (Zhang et al., 2021). The treatment successfully induced the formation of extracellular matrix fibers, especially collagen I and elastin, and smooth muscle in the vagina. Furthermore, microvascular density was increased along with more pronounced VEGF expression in the treated group (Zhang et al., 2021).

Overall, preclinical results support the use of MSCs to overcome menopausal symptoms and restore the function of the ovaries and uterus. MSC therapy might add an alternative therapeutic option to the standard hormone replacement therapy for the treatment of female reproductive diseases.

### MSC therapy for female reproductive disorders: Clinical trials

Clinical trials investigating MSC therapy in the landscape of female reproductive disorders are still in an early stage (Takahashi et al., 2021; Zhao et al., 2019; L. Chen et al., 2018). Some studies have performed stem cell injection into the uterus followed by hormone replacement therapy for women with severe Asherman syndrome or endometrial atrophy (Cao et al., 2018; Lee et al., 2020; Ma et al., 2020; Singh et al., 2020). Overall, they showed superior endometrial regeneration and higher pregnancy rates after treatment. Menstruation was restored in amenorrhea women and prolonged or increased in menstrual amounts in the other patients (Lee et al., 2020; Ma et al., 2020; Singh et al., 2020).

Furthermore, MSC therapy has been investigated for the treatment of primary ovarian failure in phase I and II trials showing improved follicular development after intraovarian injection of MSCs (L. Ding et al., 2018b; Edessy et al., 2016; Gupta et al., 2018; Herraiz et al., 2018; Igboeli et al., 2020; Mashayekhi et al., 2021; Mohamed et al., 2018; Yan et al., 2020). Estradiol was elevated after umbilical cord and bone marrow-derived MSC administration (Igboeli et al., 2020; L. Ding et al., 2018b). Reduced menopausal symptoms and return of menstruation were also commonly reported (Edessy et al., 2016; Igboeli et al., 2020; Mashayekhi et al., 2021). Two of 14 umbilical cord-derived MSC-injected patients were pregnant after years of infertility (L. Ding et al., 2018b). Gupta reported a case of a perimenopausal woman with primary ovarian failure who delivered a healthy baby after receiving autologous bone marrow-derived MSCs (Gupta et al., 2018). Mashayekhi et al. infused autologous ASCs into nine women with primary ovarian failure at three different doses of 5 × 10^6^, 10 × 10^6^, or 15 × 10^6^ cells/kg body weight (Mashayekhi et al., 2021). During the 24-months follow-up, no side effects or complications occurred. In the group receiving the highest dose, two of three patients resumed menstruation after 2 months, and menstruation was observed again 1 month after infusion in two of six patients in the other groups. Serum FSH levels decreased to less than 25 IU/l in four patients, while ovarian size did not differ between the groups (Mashayekhi et al., 2021). Recently, we performed an intravenous infusion of ASCs in 16 women with sexual hormone deficiency at a dose of 1 × 10^6^/kg body weight and followed up for 12 months after the infusion (Nguyen LT. et al., 2021). The study showed that no serious adverse events or adverse events occurred in the patients. Women reported satisfaction with their sex lives; however, there was no significant change in AMH, FSH and estradiol levels (Nguyen T. et al., 2021).

Because these studies were performed in only small numbers of patients without control groups, the power of their results remains limited. Therefore, larger randomized controlled trials will be necessary to investigate the safety and potential efficacy of MSC-based therapy in the management of female reproductive disorders.

### Potential mechanisms of ASCs for the treatment of female sexual dysfunction

**FIGURE 2:**
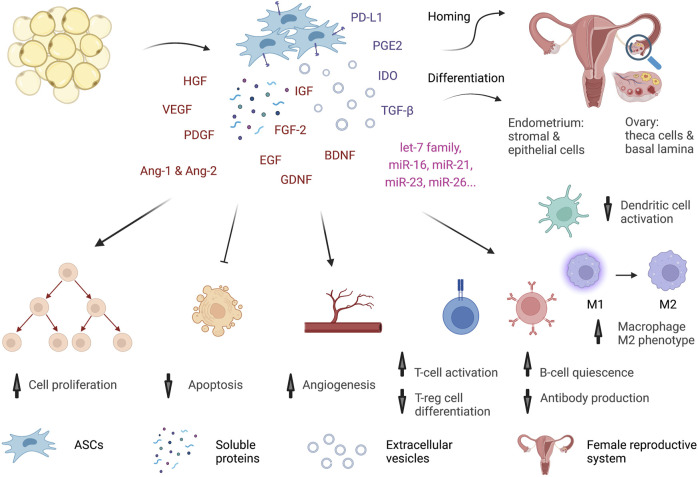
Potential mechanisms of ASCs. ASCs might act through multiple mechanisms to improve the functions of the female reproductive system. The cells can home and be retained in the reproductive tract for a short time. In the ovary, they differentiate into theca cells and form basal lamina to support oocyte development. In the uterus, ASCs give rise to stromal and epithelial cells of the endometrium. Similar to MSCs from other tissue sources, ASCs exhibit a profound secretome and immune regulation activity. The cells secrete many growth factors, cytokines, and miRNAs in the form of both soluble proteins and extracellular vesicles. Growth factors, such as HGF, VEGF, IGF, FGF-2, EGF, GFNF, and BDNF, trigger cell proliferation, inhibit apoptosis, and promote wound healing in many organs, including the reproductive system. VEGF, PDGF, HGF, Ang-1 and Ang-2 orchestrate angiogenesis. Moreover, ASCs produce many strong anti-inflammatory factors, such as PGE2, IDO, TGF-β, and PD-L1, to regulate immune cells. ASCs inhibit Th1-cell proliferation and promote regulatory T-cell differentiation. They induce quiescence of B cells and alter antibody production. They shift macrophages from a proinflammatory M1 to an anti-inflammatory M2 phenotype and interfere with dendritic cell maturation. Hence, ASCs might reverse the aging process by restoring reproductive organ functions and increasing estradiol levels, modulating the immune system, and reducing inflammation.

ASCs might act in different ways to improve sexual dysfunction in premenopausal women (Figure 2).

#### Homing, retention, and differentiation

GFP-labeled ASCs resided in the ovarian interstitial tissue surrounding oocytes 7 days after *in situ* transplantation (Terraciano et al., 2014). In concordance with this observation, GFP^+^ ASCs were retained in ovaries 14 days after injection, but most of them disappeared after 4 weeks (Su et al., 2016), while Sun *et al.* was able to detect GFP + ASCs in ovaries 1 month after both intravenous and *in situ* injection (M. Sun et al., 2013). The cells did not differentiate into oocytes or granulosa cells but rather took part in the microenvironment of these cells. Indeed, ASCs gave rise to the basal lamina and the theca layer to support granulosa cell function in a rat model of primary ovarian failure (Su et al., 2016). Similarly, ASCs and ASCs on collagen scaffolds differentiated into epithelial and stromal cells of the endometrium in an Asherman’s syndrome model with a longer retention time in the latter group (H. Sun et al., 2018). ASCs were also able to be differentiated *in vitro* into vascular endothelial cells when cultured with umbilical vein endothelial cells (Fischer et al., 2009; Bekhite et al., 2014).

#### Elevated follicle numbers and estradiol levels

Viable follicles increased in the ASC-injected group compared to the control, but estradiol concentration in serum was similar between the two groups (Terraciano et al., 2014). Fouad *et al.* reported that ASCs induced the development of ovarian follicles and yellow bodies in concordance with higher serum estradiol levels and reduced FSH (Fouad et al., 2016). Injection of ASCs with collagen scaffolds resulted in a better estrous cycle and an increase in estradiol (Su et al., 2016).

#### Paracrine effects of ASCs to promote angiogenesis and cell growth

ASCs signal to other cells through the synergistic action of soluble proteins and extracellular vesicles. ASCs are superior sources of growth factors such as vascular endothelial growth factor (VEGF), hepatocyte growth factor (HGF), fibroblast growth factor 2 (FGF-2), epidermal growth factor (EGF), platelet-derived growth factor (PDGF), angiopoietin-1 and -2 (Ang-1 and Ang-2), brain-derived neurotrophic factor (BDNF), glial cell-derived neurotrophic factor (GDNF), and insulin-like growth factor (IGF) (Rehman et al., 2004; Sadat et al., 2007; Kingham et al., 2014; Dabrowski et al., 2017; Y.-H. An et al., 2021; Jiao et al., 2021; Ngo et al., 2021; Sumarwoto et al., 2021). These factors mediate ASCs to promote angiogenesis (Jiao et al., 2021; Krawczenko and Klimczak 2022), cell survival (Rehman et al., 2004), cell proliferation (Jiao et al., 2021), and wound healing processes (Y.-H. An et al., 2021) in many disease models. The role of growth factors produced in the reproductive system was reviewed previously, suggesting a complex regulated network of signaling pathways orchestrated in ovarian development and function (Evron et al., 2015). IGF signaling is essential for follicular development (Giudice 1992), in which IGF-1 stimulates the proliferation of human granulosa cells and induces estradiol and progesterone production in the ovary (Zhou et al., 2013). It also cooperates with FSH to regulate germ cell differentiation (Zhou et al., 2013). FGF-2 accompanied by progesterone maintains the survival of granulosa cells and epithelial cells on the surface of ovaries (Trolice et al., 1997; Peluso and Pappalardo 1999). FGF-2 and EGF can stimulate granulosa cell proliferation (Gospodarowicz and Hugh 1979). VEGF and neurotrophic factors, such as BDNF and GDNF, also protect follicular cells from apoptosis and therefore regulate oocyte maturation (Shin et al., 2006; Linher-Melville and Li 2013). VEGF plays a multipotent role during follicular development. Inhibition of VEGF-A interfered with theca and granulosa cell proliferation, diminished follicular angiogenesis, disrupted ovulation, and led to miscarriage (Wulff et al., 2001; Fraser et al., 2010). In a mouse model of vaginal atrophy, ASC administration induced increased expression of VEGF and its receptor in both connective tissue and the epithelial layer of the vagina in correlation with a higher anti-apoptotic factor bcl2 and reduced epithelial damage (Kasap et al., 2019).

In addition to soluble proteins, extracellular vesicles are another form of cell communication (Mittelbrunn and Sánchez-Madrid 2012; Turturici et al., 2014). Their cargos include lipids, growth factors, cytokines, membrane proteins, and genetic materials, especially microRNAs (Zhang et al., 2019; Wei et al., 2021). Mitchel et al. identified more than 20,000 miRNA sequences of the ASC secretome and extracellular vesicles, of which half of the target mRNAs were linked to signal transduction, response to stress, and regulation of cell differentiation and proliferation (Mitchell et al., 2019). The proangiogenic let-7 family, miR16, miR-23a, and miR-23b were highly expressed in their analysis (Landskroner-Eiger et al., 2013; Mitchell et al., 2019). Overexpression of mRNA-21, which is a key regulator of cancer angiogenesis, enhanced vascularization by upregulating HIF-1α, VEGF, Akt and Erk signaling (Y. An et al., 2019). Moreover, miR-21 activated the PI3K/AKT signaling pathway to promote cell migration and proliferation during the wound healing process (C. Yang et al., 2020).

#### Immunomodulatory potential of ASCs

ASCs demonstrate a superior immunomodulatory capacity compared to MSCs from other tissue origins, such as bone marrow, dental pulp, and umbilical cord (Melief et al., 2013; Ribeiro et al., 2013; Mattar and Bieback 2015). The cells regulate the innate and adaptive immune systems through both direct cell‒cell contact and secretion of cytokines and other soluble factors (Al-Ghadban and Bunnell 2020; Ceccarelli et al., 2020). ASCs secrete many anti-inflammatory factors, including prostaglandin E2 (PGE2), indoleamine-2,3-dioxygenase (IDO), transforming growth factor-beta (TGF-β), and programmed cell death ligand (PD-L1) (J.-H. An et al., 2018; Bulur and Dietz 2018; Cho et al., 2015; Eljaafari et al., 2021; Kawada-Horitani et al., 2022; Mckinnirey et al., 2021; Yañez et al., 2010; Zheng et al., 2017). The cell also produces highly anti-inflammatory miRNAs, especially the let7 family, miR-26a-5p and miR-16-5p, which are involved in immune cell regulation (Mitchell et al., 2019; Ragni et al., 2019).

ASCs inhibited Th1-cell proliferation and downregulated the expression of proinflammatory cytokines such as TNF-α, IFN-γ, and IL-12, while they stimulated regulatory T-cell differentiation (Yañez et al., 2006; Gonzalez-Rey et al., 2010; Engela et al., 2013). ASCs also have multiple effects on B cells, as they induce the quiescence of B cells, inhibit the formation of plasmablasts and activate IL-10-expressing regulatory B cells (Franquesa et al., 2015; Peng et al., 2015). Consequently, IgM, IgG, and IgA production was significantly impaired in the presence of ASCs (Corcione et al., 2006). Therefore, ASCs have been successfully applied to treat graft-versus-host disease, which is caused by the cytotoxicity of donor-derived T cells against recipient and autoimmune diseases and is mediated by B-cell misdirection toward the patient’s own cells (González et al., 2009; Peng et al., 2015; Panés et al., 2016; Fernández et al., 2018; Castro et al., 2020).

ASCs can shift macrophages from the M1 phenotype to the M2 phenotype via their secretomes, such as PGE2, IL6, TSG-6, and miR-451 (Song et al., 2017; C.-Y. Yang et al., 2021; R. Li et al., 2022; Yuan et al., 2022). M2 macrophages secrete immunosuppressive and anti-inflammatory cytokines IL-10 and TSG-6 to modulate immune reactions and activate tissue repair (Kim and Hematti 2009; Heo, Choi, and Kim 2019; R. Li et al., 2022). Similar to macrophages, dendritic cells are also a target of ASCs. A coculture of these cells resulted in blockade of dendritic cell maturation and changed them into an anti-inflammatory phenotype with enhanced phagocytosis (Anderson et al., 2017; Ortiz-Virumbrales et al., 2020). ASCs have been shown to be superior in suppressing dendritic cells compared to their bone marrow-derived counterparts (Ivanova-Todorova et al., 2009; Zaza et al., 2019). On the other hand, the interaction between MSCs, including ASCs, and NK cells is complex and remains controversial depending on the coculture conditions and prestimulation of NK cells. There are studies showing that ASCs were able to alter NK functions (DelaRosa et al., 2012; Najar et al., 2019). However, they are less potent in inhibiting NK-cell cytotoxic activity than bone marrow-derived MSCs (Valencia et al., 2016; Najar et al., 2019).

### Study protocol for a randomized controlled phase II clinical trial to investigate the therapeutic potential and mechanisms of autologous ASCs in female sexual dysfunction

**FIGURE 3:**
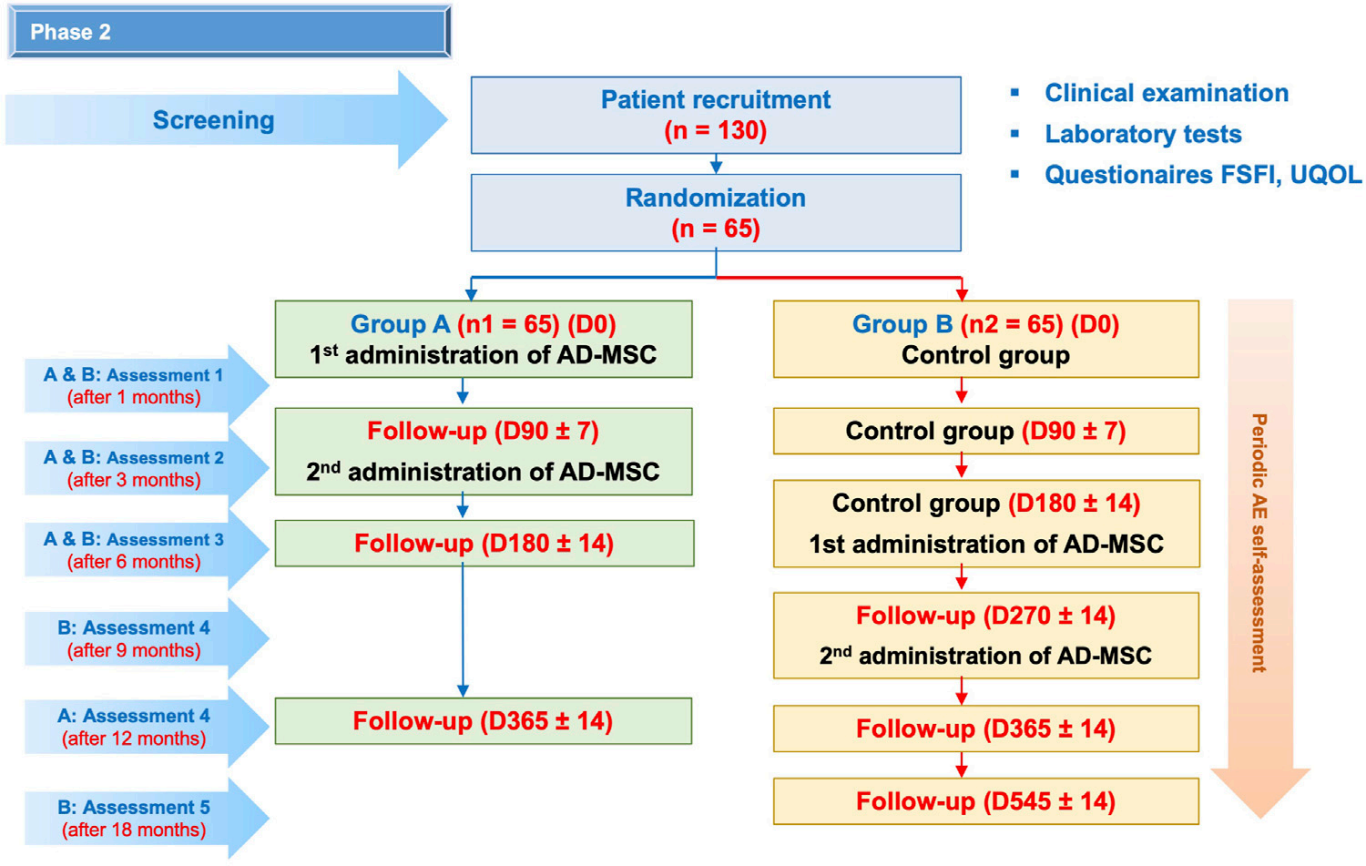
Research diagram. A total of 130 enrolled patients will be randomly assigned to two groups: group A will receive two infusions of autologous ASCs at day 0 and day 90 ± 7; group B will receive two infusions of autologous ASCs at day 180 ± 14 and day 270 ± 14. All patients will be monitored for 12 months after the first cell infusion. During this period, AEs will be recorded every week after the ASC infusions and every month in the next months by the study team. Patient visit schedules are depicted, in which physical examination, laboratory tests and FSFI and UQOL questionnaires will be administered. Furthermore, cytokine profiles will be analyzed at baseline and on days 30 ± 2, 90 ± 7, and 180 ± 14. Cellular aging biomarkers will be quantified at baseline and at 180 ± 14 and 365 ± 14.

This is a randomized controlled phase II clinical trial with a crossover design to evaluate the safety and potential efficacy of autologous ASC therapy to treat sexual dysfunction in females. A total of 130 female patients with sexual dysfunction will be recruited at the Regenerative Medicine Department at Vinmec Times City International Hospital, Hanoi, Vietnam, between September 2022 and December 2024. The inclusion and exclusion criteria of the study are presented in detail in the supplementary information. Enrolled patients will be randomly divided into two groups (Figure 3). Patients in group A will receive two infusions of autologous ASCs at day 0 and day 90 ± 7, while those in group B will be followed-up for 180 ± 14 days and then receive two infusions of autologous ASCs at day 180 ± 14 and day 270 ± 14.

Autologous ASCs used in this study were harvested from autologous fat tissue through an enzyme method and expanded using our in-house optimized serum-free and xeno-free culture under a physiological oxygen concentration of 5% as described in the supplementary information (Hoang et al., 2020). For clinical use, cells at passage 3 will be harvested in 100 ml Ringer’s lactate. Therapeutic ASC products must pass the following releasing criteria: 1) negative for bacteria, fungi and *mycoplasma*; 2) more than 90% viability; 3) expression of more than 95% positivity for CD105, CD73, and CD90 and less than 2% positivity for CD45, CD34, CD11b, CD19, and HLA-DR; and 4) endotoxin < 5 EU/kg. The patient will be infused with 1 × 10^6^ cells/kg body weight within 60 min via the intravenous route.

The patients will be monitored within 12 months after the first cell infusion (Table 1). Here, the authors use a comprehensive study plan to evaluate the efficacy of autologous ASC therapy in the management of female sexual dysfunction through three levels: 1) self-assessment questionnaires; 2) female sex hormones; and 3) biomarkers of inflammation and cellular senescence.

**TABLE 1:** Study timeline and clinical procedures during the trial.

Study procedure	Prescreening (If applicable)	Screening phase*	Baseline	Day 30 ± 3	Day 90 ± 7	Day 180 ± 14	Day 270 ± 14	Day 365 ± 14	Day 365 ± 14	Day 545 ± 14
ASC Infusion										
Group A			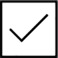		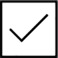					
Group B						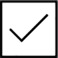	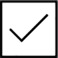			
Concomitant therapy^**^			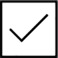	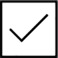	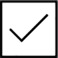	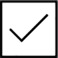	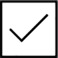			
Informed consent		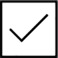								
Inclusion and exclusion criteria		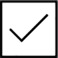								
Demographic information		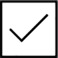	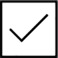							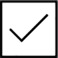
Patient’s medical reports		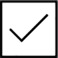	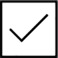	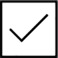	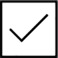	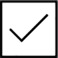	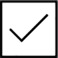	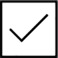	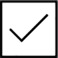	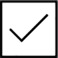
Physical examination		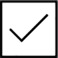	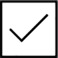	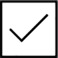	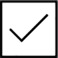	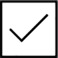	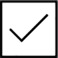	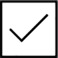	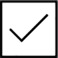	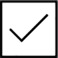
Female sex hormone evaluation (FSH, E2)	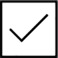	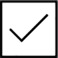	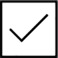		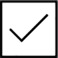	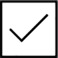	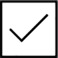	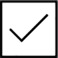	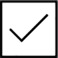	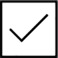
Cytokines and aging biomarkers evaluation^#^			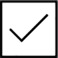	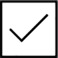	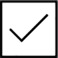	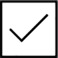	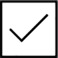			
Hematology analysis^***^	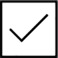	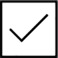	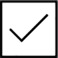		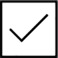	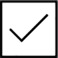	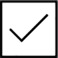	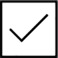	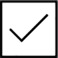	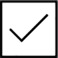
Infectious disease examination/test^##^	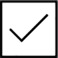	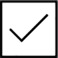	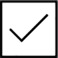							
Thrombotic analysis^§^			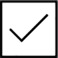	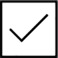	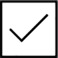	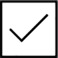	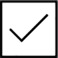	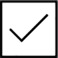	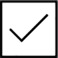	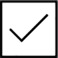
Quality of life evaluation^†^	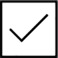	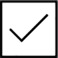	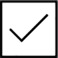	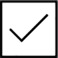	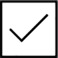	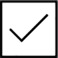	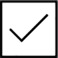	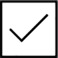	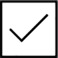	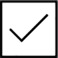
Adverse events (AEs/SAEs) evaluation			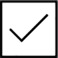	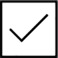	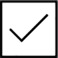	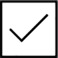	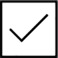	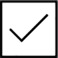	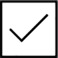	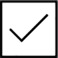
Monitoring of mortality/complications			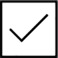	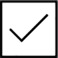	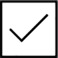	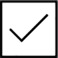	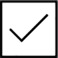	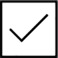	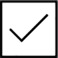	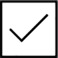
Blood sample for molecular and cellular analyses of sexual function impairment			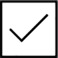	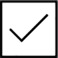	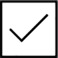	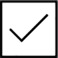	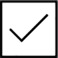	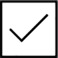	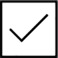	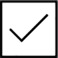

**Notes:** ASC, autologous adipose-derived mesenchymal stem/stromal cell, AEs, adverse events; SAEs = serious adverse events; FSH, Follicle-Stimulating Hormone, E2 = Estradiol Hormones, FSFI, Female Sexual Function index; HBV, hepatitis B virus, HIV, human immunodeficiency virus; UQOL , Utian quality of life Scale.

^*^If the results of the screening phase for ASC, groups are within 30 days of ASC, administration, they will be automatically considered as the baseline level.

^**^The concomitant therapy administered to both groups included Hightamin, total Calcium, Bioflex, Cic-Zinc.

^§^The thrombotic analysis included measurement of the D-dimer, fibrinogen, prothrombin, thrombin, and APTT, levels prior to ASC, administration and 24-h post-administration.

^***^The hematological analysis included measurements of the white blood cell count, platelet count, red blood cell count, hemoglobin, and percentages of lymphocytes, neutrophils, monocytes, eosinophils, basophils, C-reactive protein, pro-BNP, and troponin-T.

^#^Blood samples will be collected for cellular and molecular analysis, including analyses of cytokines, chemokines, and aging biomarkers in the patient’s plasma: TNFa, IFN-γ, IL1, IL-6, IL-8, IL-4, IL-10, and IDO, plasminogen activator inhibitor-1 (PAI-1), p16 and p21 expression.

^##^Infectious diseases include hepatitis, syphilis, HIV, HBV, and *tuberculosis*.

^†^ The quality of life evaluation includes the self-assessment questionnaires: FSFI and UQOL.

The improvement of sexual function and overall quality of life will be assessed through the FSFI and the Utian Quality of Life Scale (UQOL). The FSFI is a self-assessment questionnaire to quantify female sexual function. The FSFI index has high to very high reliability and repeatability (Rosen et al., 2000; Meston 2003; Wiegel et al., 2005), and a score of ≤ 26.55 is classified only as female sexual dysfunction (FSD) (Wiegel et al., 2005. The UQOL is a modern instrument to measure the quality of life of menopausal women (Utian et al., 2018). It has been clinically proven effective and has been applied across many countries (Abay and Kaplan 2016; Balanian et al., 2020; Moravcova et al., 2022).

Furthermore, changes in the levels of FSH and estradiol will be examined. FSH stimulates granulosa cells in ovarian follicles to synthesize aromatase, which converts androgens produced by thecal cells to estradiol (Steinkampf et al., 1987; Hillier et al., 1994). Estradiol is a steroid hormone associated with the female reproductive organs and is responsible for the development of female sexual characteristics (Findlay et al., 2010). Elevated FSH and decreased estradiol concentrations are responsible for menopausal symptoms, including decreased functions of reproductive organs (Mason 1976). Therefore, levels of FSH and estradiol would reflect female sexual health in general and during the perimenopausal period.

To study ASC effects at the cellular and molecular levels, the serum concentrations of cytokines (TNFa, IFN-γ, IL1, IL-6, IL-8, IL-4, IL-10, IDO) will be quantified. In addition, the expression of senescence biomarkers, including plasminogen activator inhibitor-1 (PAI-1), p16, and p21, is also of interest. Since chronic inflammation and the natural aging process increase the incidence of female sexual dysfunction, the analysis might provide a hint for the mechanisms of action of cell therapy.

### Rationales of the crossover design of the proposed clinical trial

A randomized crossover trial is a prospective study in which participants received two or more sequential interventions in a random order, often separated by a washout period (Senn 2002). It allocates participants to different treatments over two or more periods, while in a parallel trial, participants are randomized to the same intervention over a single period (Mills et al., 2009).

In this study, we choose a two-sequence crossover design since the AB/BA design has some advantages over the parallel-group design. First, the two-sequence crossover design is robust and applicable when there is considerable between-patient variability and less within-patient variability (Gewandter et al., 2019). Second, fewer participants needed to be recruited for similar statistical power (Senn 2002; Chow and Liu 2009; Wellek and Blettner 2012). A crossover trial is a “within-subject” study design where each participant acts as his or her control (Sedgwick 2014). Crossover trials may offer more precise intervention effect estimates than parallel trials because they would remove any biological and methodological variation (Mills et al., 2009).

However, the crossover design also has some weaknesses. The time to conduct a crossover trial is longer than that to conduct a parallel-group trial. Moreover, the cross-sectional design may even induce bias, such as confounding, which can also arise from sequential randomization of an insufficient number of clusters (Goldstein et al., 2018) or the effects of attrition (Moerbeek 2020). In addition, proper statistical methods are required to analyze crossover trials to reduce treatment effects, carryover effects, and period effects (Nason and Dean 2010; Wellek and Blettner 2012; Sturdevant and Lumley 2021).

## Discussion

### Potential side effects of ASC therapy in general medicine including female sexual dysfunction

A meta-analysis investigated adverse events after MSC administration based on 62 prospective studies with the longest follow-up of 5 years (Wang et al., 2021). There was no connection between the therapy and cancer and mortality incidence. The most common major side effect was transient fever, which developed within 48 h after cell administration, followed by administration site adverse events such as bleeding, swelling, pain, itching and infection at the injection site. Minor side effects that were associated with MSC therapy included sleeplessness, fatigue, and constipation. Other events, such as seizure, vomiting, anemia, and nausea, were significantly associated with the therapy. Moreover, AD-MSCs more frequently caused headache and dizziness than bone marrow-derived MSCs (Wang et al., 2021). An update of the largest phase 3 randomized, double-blind control trial investigating the safety and efficacy of a single local administration of allogenic ASCs (Cx601) in patients with Crohn’s disease and perianal fistulas was reported (Panés et al., 2018). Although the treatment group experienced more frequent serious treatment-emergent adverse events during the 52-weeks follow-up than the control group (24,3% versus 20,6%, respectively), treatment-related serious events were comparable (6.8% versus 6.9%, respectively). The events included anal abscess/fistula in the treatment group and anal abscess/fistula, proctalgia, anal inflammation, and liver abscess in the control group (Panés et al., 2018).

The administration of allogenic MSCs might trigger immune reactions and immune rejection. This could be a cause of transient fever observed in patients after treatment (Wang et al., 2021). On the other hand, because ASCs express only low levels of major histocompatibility complex (MHC) class I molecules and are absent in MHC class II and costimulatory molecules, including CD40, CD80, and CD86, their immunogenicity remains low (L. Cui et al., 2007). Their strong immunosuppressive capacity further favors these cells to escape the host immune system (Yañez et al., 2006). In the Cx601 phase trial, no immune reactions or adverse events related to the development of donor-specific antibodies were observed. On the other hand, the ASC-treated group had a higher risk of developing IgG HLA class I antibodies, with a rise from 16% at baseline to 34% at week 12, in contrast to no change in the control group. The presence of donor-specific antibodies did not correlate with treatment outcome (Panés et al., 2016).

ASCs might activate coagulation when exposed to blood and increase the risk of thrombosis in patients undergoing stem cell therapy (Jung et al., 2013). Pulmonary embolism and infarct in the right lung were observed in a patient after being intravenously infused with three doses of autologous ASCs to treat the cervical herniated intervertebral disc. His parents, who received a similar therapy for knee osteoarthritis, also developed multiple embolisms in both pulmonary artery branches with right pleural effusion (Jung et al., 2013). The use of anticoagulants such as heparin and EDTA is recommended to prevent thrombogenesis (Liao et al., 2017; Moll et al., 2019).

A link between ASC therapy and tumorigenesis is still under investigation. Preclinical data suggest that ASCs can activate pathways involved in tumor formation and progression (Sabol et al., 2019). In most scenarios, ASCs interacted with the tumor microenvironment and mediated cell growth, metastasis, and chemoresistance of tumor cells in experimental breast, ovarian, cervical cancers, etc. (Zhang et al., 2015; Goto et al., 2019; Castro-Oropeza et al., 2020). However, clinical data supporting a connection between MSC treatment and tumor development are still lacking. In patients with hematologic malignancy, cotransplantation of bone marrow-derived MSCs with hematopoietic stem cells reduced graft-versus-host disease severity; however, it was associated with a higher relapse rate and increased patient mortality over a 3-year observation *period* (Ning et al., 2008). As a balance between graft-versus-leukemia and graft-versus-host reactions is required for a durable response of transplant patients, it remains unclear whether the disease recurrence was due to lower anti-leukemia activity of graft-derived T cells or MSC-induced leukemia stimulatory effects (Munker et al., 2004; Munker and Kolb., 2006).

Overall, clinical data suggest that ASC therapy is well tolerated with considerable side effects. However, long-term observations, especially in terms of tumorigenesis and the identification of risk factors/risk populations, remain elusive.

### Using autologous versus allogenic ASCs: pros and contras

MSCs are suitable both in an autologous and an allogenic setting. Autologous cell transplantation is generally safer. There is no concern of infectious disease transmission and host versus graft incompatibility (Kot et al., 2019). Although MSCs express only a limited level of MHC class I and lack MHC class II and its cofactors (Samadi et al., 2021; J. Chen J-m et al., 2021), inflammatory reactions, graft rejection, and the development of graft-versus-host disease have been reported in animal models (Poncelet et al., 2007; Pezzanite et al., 2015; Owens et al., 2016). The choices between autologous versus allogenic cells often depend on the disease background, availability of cells (and donors), cost and window of delivery. Many factors can negatively impact MSC quality. Cells exhibit altered cellular functions leading to decreased regenerative bioactivity with increasing age (Peffers et al., 2016; Marędziak et al., 2016; Y.-H. K. Yang 2018). In addition, the authors have demonstrated that long-term type II diabetes mellitus could induce remarkable changes in mtDNA genetic profiles and negatively interfere with cell metabolism and bioactivity (Nguyen T. et al., 2021). On the other hand, potential genetic changes in the reproductive system need to be considered when allogenic cells are applied for the management of reproductive health (X. Chen et al., 2021b). Thus, challenges such as the maintenance of healthy MSC sources and boosting of their potency remain to be addressed in the case of autologous cell therapy, while immune reactions, graft rejection, and ethical concerns are of interest for allogenic use.

### Enhancing the therapeutic activity of ASCs via hypoxic culture conditions

Physioxia (also known as hypoxia compared to the ambient oxygen concentration of the atmosphere) is a promising strategy to accelerate MSC functions both *in vitro* and *in vivo*. Under this condition, MSCs maintained a longer undifferentiated state (Basciano et al., 2011) and a longer proliferative lifespan before reaching senescence (Grayson et al., 2006). Furthermore, physioxia enhances MSC secretion of proangiogenic factors such as VEGF, IGF, HGF, and bFGF, as well as the immunomodulatory molecule TGF-β (Kinnaird. et al., 2004; Crisostomo et al., 2008; Ranganath et al., 2012; Noronha et al., 2019). In animal models, physioxia-cultured human MSCs demonstrated enhanced *in vivo* survival (Beegle et al., 2015). Physioxia successfully boosted MSC potencies in the treatment of ischemia (Han et al., 2016; Noronha et al., 2019) and lung damage induced by radiation or bleomycin (Lan et al., 2015; B. Li J. et al., 2017) compared to those cultured in ambient oxygen concentration. Thus, physioxia culture might improve the therapeutic potential of MSCs. However, implementation of physioxia culture in clinical settings remain to be investigated.

## Summary

ASCs are a potent candidate for cell therapy with high growth factor secretion activity and superior immunomodulatory capacity. The side effects of ASC therapy are manageable, and there was no connection between the treatment and incidence of cancer and mortality in treated patients. However, longer follow-ups are required to study late events, especially in tumor formation and progression. ASC therapy for female reproductive diseases has been investigated preclinically and in some phase I clinical trials, suggesting a potential activity of ASCs in female sexual dysfunction. Based on our current knowledge about the benefits and challenges of ASCs, the authors have introduced the design of our phase II trial to study the safety and efficacy of autologous ASCs in the management of sexual dysfunction in perimenopausal women. The results of the proposed study will provide profound insight into MSC actions in this disease. It might also encourage a transition of MSC culture from high to physiological oxygen conditions in future research.
