# Supplementary material for: “Adipose-derived mesenchymal stem cell therapy for the management of female sexual dysfunction: Literature reviews and study design of a clinical trial”
Source: Front Cell Dev Biol. 2022 Sep 28;10:956274. doi: 10.3389/fcell.2022.956274 (PMC9554747; doi:10.3389/fcell.2022.956274)
Supplement: Supplementary file 1 [file DataSheet1.docx]

**Supplementary information**

**Title: “Adipose-Derived Mesenchymal Stem Cell Therapy for the Management of Female Sexual Dysfunction: Literature Reviews and Study Design of a Clinical Trial"**

Van T. Hoang^1^, PhD., Hoang-Phuong Nguyen^1^, Viet Nhan Nguyen^2,3^, Duc M. Hoang^1^, PhD., Tan-Sinh Thi Nguyen^2^, Liem Nguyen Thanh^1,2,3^, PhD. MD.

*^1^ Vinmec Research Institute of Stem Cell and Gene Technology, Vinmec Health Care System,* *458 Minh Khai, Hanoi 11622, Vietnam*

*^2^ Vinmec International Hospital – Times City, 458 Minh Khai, Hanoi 11622, Vietnam*

*^3^ College of Health Science, Vin University, Vinhomes Ocean Park, Gia Lam District, Hanoi 12400, Vietnam*

**Corresponding authors:**

Liem Nguyen Thanh, PhD. MD., Vinmec Research Institute of Stem Cell and Gene Technology, 458 Minh Khai, Hai Ba Trung District, Hanoi, Vietnam. Phone: (+84 24) 3 975 1418. Email: [v.liemnt@vinmec.com](mailto:v.liemnt@vinmec.com);

Tan-Sinh Thi Nguyen, MD., Vinmec International Hospital – Times City, Vinmec Health Care System, 458 Minh Khai, Hai Ba Trung District, Hanoi, Vietnam. Phone: (+84 24) 3 975 1418. Email: v.sinhntt@vinmec.com

**Key words:** sexual function impairment, female sexual dysfunction, AD-MSCs, stem cell therapy, regenerative medicine, menopause

***Study objectives***

This trial aims to evaluate the safety and potential efficacy of autologous AD-MSC therapy to treat sexual function impairment in females.

The primary objectives are as follows:

- We compared the improvement in sexual function and overall quality of life at the time points before the intervention and 1 month, 3 months, 6 months and 12 months after the first AD-MSC transfusion through the self-assessment questionnaires: the Female Sexual Function Index (FSFI) and the Utian Quality of Life Scale (UQOL).
- The safety of AD-MSC therapy was investigated based on the occurrence and severity of adverse events (AEs) and serious adverse events (SAEs) up to the end-of-study visit on day 365 ± 14 after the first AD-MSC transfusion.

The secondary objectives include the following:

- The levels of FSH and estradiol before the intervention and 1 month, 3 months, 6 months and 12 months after the first AD-MSC infusion were compared.
- Evaluating the levels of cytokines and aging biomarkers:
- Assessing inflammation of the body through the concentration of cytokines (TNFa, IFN-γ, IL1, IL-6, IL-8, IL-4, IL-10 and IDO) in plasma at the time of intervention and 1 month, 3 months and 6 months after the first AD-MSC infusion.
- We analyzed aging biomarkers, including plasma expression of plasminogen activator inhibitor-1 (PAI-1), p16 and p21, at the time of intervention and 6 months and 12 months after the first AD-MSC infusion.

***Study design and approval***

This is a randomized controlled phase II clinical trial with a crossover design. A total of 130 female patients with sexual function impairment will be recruited at the Regenerative Medicine Department at Vinmec Times City International Hospital, Hanoi, Vietnam, between September 2022 and December 2024. The enrolled patients will be randomly divided into two groups as described in Figure 2.

- Group A: patients will receive two infusions of autologous AD-MSCs at day 0 and day 90 ± 7.
- Group B: patients will be followed-up for 180 ± 14 days and then receive two infusions of autologous AD-MSCs at day 180 ± 14 and day 270 ± 14.

Both groups will be monitored within 12 months after the first cell infusion.

The study was approved by the Ethical Committee of Vinmec International Hospital (number: 30/2022/CN- HDDD VMEC). This study was registered at ClinicalTrials.gov (number NCT05329662).

***Sample size calculation***

In our phase I study, the mean of the Female Sex Function Index (FSFI) at baseline was 20.66 points with a standard deviation of 6.99. At 12 months after the intervention, the FSFI index reached 27.12 points with a standard deviation of 3.92. Therefore, we expect the FSFI index to increase to 25.0 points after 12 months of intervention for this study. According to the continuous endpoint of two independent sample studies, we assumed α was 0.05 and that the power was 90%. Thus, the smallest sample size was 130 patients, with a dropout rate of 15%. Therefore, the calculated sample size was 65 for each group.

***Randomization***

All patients will be randomized (1:1) into either group A (n=65) or group B (n=65) (Supplementary Figure 1). Patients from both groups will receive a standard medication treatment according to the Vietnamese Ministry of Health guidelines, which includes the use of Hightamin, total Calcium, Bioflex, and Cic-Zinc as a supplementary medication in three months.

***Study population***

*Inclusion criteria:*

- Women who are 40 - 50 years of age
- Not yet menopause (at stage -3a, -2, -1 according to the 2011 Stages of Reproductive Aging Workshop criteria STRAW + 10 (Harlow et al. 2012; Randolph et al. 2011).
- FSH level:
  - ≤ 21,9 IU/L và >2,6 IU/L
  - The FSH blood test was performed on days 2-7 of the menstrual cycle (in the morning).
- FSFI score (Female Sexual Function Index) < 26.55 or AMSDM score (AMS Diagnosing Menopause: Symptom Score Sheet) ≥ 15
- Normal liver function
- Normal kidney function
- Normal cardiovascular function
- No active bacterial, fungal or viral (HIV, HBV, HCV, syphilis) infections
- Provide written informed consent

*Exclusion Criteria:*

- Previous surgery to remove gonads
- Without adipose tissue
- Spinal cord injury
- No sexual activity
- Diagnosis of cancer
- Had an organ transplant
- Had congenital malformations related to the gonads
- Diagnosed with chronic diseases such as diabetes, hypopituitarism, adrenal insufficiency, and blood pressure unresponsive to treatment
- Diagnosis of active autoimmune diseases
- Diagnosed with heart failure, kidney failure, liver failure, respiratory failure, history of cerebral infarction, myocardial infarction, Alzheimer's
- Hypothyroidism or hyperthyroidism
- Diagnosed with clinically significant blood coagulation disorders.
- History of allergic reaction to anesthetic agents and antibiotics
- Using hormone therapy within the last two weeks or wanting to use these drugs during the study period
- Planning to become pregnant during the study period
- Taking birth control methods that involve systemic hormones
- Absence of menstruation for at least 12 months
- Mental illness, inability to communicate, inability to answer the interview questions

***Patient recruitment***

The patients diagnosed with sexual function impairment will be approached and asked to participate in the study at either Hanoi Medical University Hospital (Hanoi, Vietnam) or Vinmec International Hospital Times City (Hanoi, Vietnam). If the patients are interested in this research, they will be asked to send prescreen results to a study doctor. The doctor will consult with the potential candidates about the study, including the clinical trial process, the advantages and disadvantages of autologous AD-MSC therapy, and the potential adverse events. The patients can only be enrolled in this trial after completing the consultation and signing the informed consent form.

***Withdrawal***

Participant discontinuation may occur upon participant death, severe adverse events (SEAs), other serious disease-limiting involvement, or a direct request from participant to withdraw from the study. Once the participant withdraws from the study, the reasons for the withdrawal and all recorded results will be documented in detail. New participants will not be recruited to replace withdrawn participants.

***Study agent***

**Isolation and culture of autologous AD-MSCs**

Liposection will be performed under local anesthesia in an operating room to collect from 50 to 150 mL fat. The samples will be further processed in an ISO 14644-1, class 6 clean room to isolate AD-MSCs. The cells were isolated by the enzyme method, in which aspirated fat tissue was digested in a 200 unit/ml collagenase type I solution (Gibco, Denmark) supplemented with 200 g/l human albumin (Baxter, USA) at 37 °C. The stromal vascular fraction was collected in StemMACS™ MSC Expansion Media (Miltenyi Biotec, Germany) and cultured in 2% O_2_ under optimized serum-free and xeno-free conditions as described previously (Hoang et al. 2020). Cells were passaged after reaching 80–90% confluence using TrypLE™ Select Enzyme (Gibco, USA). A stock of the cells was banked at passage 0 in Cryostor solution (Stemcell Technologies, Canada) in a BioStore™ III Cryo (Brooks Life Sciences, USA) for subsequent uses. For infusion into patients, AD-MSCs were cultured in StemMACS™ MSC Expansion Media until passage 3. On the day of transplantation, the AD-MSCs were harvested, washed with 0.9% NaCl, and counted for cell number and viability. Cells were resuspended in 100 mL ringer lactate and tested for batch release criteria before delivery to an operating room.

**Quality control of the AD-MSC product**

**After isolation,** AD-MSCs will be evaluated for quality according to the International Society for Cell & Gene Therapy (ISCT) and FDA recommendations for MSC-based products as follows (Bourin et al. 2013; Dominici et al. 2006; Mendicino et al. 2014):

- Sterility test: test for mycoplasma, bacteria and fungi

- Identity test: Quantifying the surface expression of markers including CD105, CD73, CD90, CD45, CD34, CD11b, CD19, HLA-DR and CD31 by flow cytometry

- Bioactivity test:

- Assessing the number of cells at passage 0, the doubling time and the viability rate of cells from passage 0 to 3
- Analysis of colony-forming capacity at passage 3
- Investigating the capacity to differentiate into osteocytes, adipocytes and chondrocytes at passage 3

- Safety:

- Expression of the pro-coagulation factor CD142

- Potency test:

- Secretion profile
- Immunoregulation of T cells

**Releasing criteria of cell products**

AD-MSCs need to fulfill the release criteria described in Table 1.

Table 1: Releasing criteria of AD-MSCs

| **Category** | **Requirements of intravenously infused MSC products** |
| --- | --- |
| Numbers of AD-MSCs | 10^6^ cells/kg of weight |
| Viability | >70% |
| Endotoxin | <5 EU/kg |
| Bacteria | Negative |
| Fungi | Negative |
| Mycoplasma | Negative |
| MSC markers | > 95% positivity for CD105, CD73, and CD90  < 2% positivity for CD45, CD34, CD11b, CD19, HLA-DR and CD31 |

***Intervention***

**Cell administration**

Within 7 days before each cell administration appointment, a study doctor will examine the participant and perform laboratory tests, including CBC, coagulation, D-dimer, estradiol and FSH levels. On the day of cell infusion, the patient will receive Solumedrol 40 mg/1 intravenously. After 30 min, she will then be infused with 1x10^6^ cells/kg body weight in 100 mL ringer lactate within 60 minutes via the intravenous route. On the next day, coagulation and D-dimer tests will be performed to check for potential instant blood-mediated inflammatory reactions. The patients will be monitored for 24 hours and discharged unless adverse events occur.

All patients in the two groups will receive two doses of autologous AD-MSCs at a 3-month interval.

- - Group A received the first infusion of AD-MSCs from autologous adipose tissue on day 0 and the second infusion on day 90±7 and was then followed up for 12 months after the first infusion. Participants will be assessed at baseline and on days 30±7, 90±7, 180±14 and 365±14.
  - Group B was followed up in the first six months and then received AD-MSCs from autologous adipose tissue the first time on day 180±14 and the second time on day 270±14. Participants will be followed up for up to 12 months after the first infusion. Participants will be assessed at baseline and on days 30±7, 90±7, 180±14, 270±14 and 545±14.

Concomitant therapy: Both groups received vitamins, including higamin, total calcium, bioflex, and cic-zinc, for 30 days after screening and after the first and second AD-MSC infusions. The total duration was three months.

***Outcome measurement***

**Primary outcome (efficacy)**

- Changes in sexual functions and overall quality of life at the time points before the intervention and 1 month, 3, 6 and 12 months after the first AD-MSC transfusion based on the Self-Assessment Self-Assessment Questionnaire, Female Sexual Function Index (FSFI) and UTIAN Quality of Life Scale (UQOL: The Utian Quality of Life Scale).

The FSFI is a self-assessment questionnaire to quantify female sexual function. The FSFI index has high to very high reliability and repeatability (Meston 2003; Rosen, C. Brown, J. Heiman, S. Leib 2000; Wiegel, Meston, and Rosen 2005). The FSFI questionnaire consists of 19 questions related to sexual activity within the 4 weeks prior to the examination. Points are assigned for each answer (1-5 and 0-5 for questions 1-2 and questions 3-19, respectively), the sum of the scores for the domain is multiplied by the domain factor, the six domain scores are summed, and the total score may vary from 2.0 to 36.0 points. A score of ≤ 26.55 is classified only as female sexual dysfunction (FSD) (Wiegel, Meston, and Rosen 2005).

UQOL has been clinically proven effective in assessing quality of life in perimenopausal women (Utian et al. 2018). The instrument contains 23 questions regarding four components of quality of life, including (1) occupational, (2) health, (3) emotional, and (4) sexual quality of life. Items are scaled using a Likert-type 1 to 5 rating resulting in a maximal total score of 115.

***Primary outcome (safety)***

To evaluate the safety of autologous adipose tissue-derived mesenchymal stem cell (AD-MSC) therapy to treat sexual function impairment in females, the number of adverse events (AEs) and serious adverse events (SAEs) during stem cell administration and throughout the follow-up will be recorded. The common AEs and SEAs of the administration of autologous AD-MSCs were previously described (Chung et al. 2021; Mohamed et al. 2020; Nguyen Thanh et al. 2021), including death, thromboembolic events, stroke, cardiovascular abnormalities, clinically significant laboratory test abnormalities, and thrombotic consequences.

**Secondary outcome (efficacy)**

- Changes in the levels of follicle-stimulating hormone (FSH) and estradiol at the time points before the intervention and 1 month, 3 months and 6 months after the first AD-MSC infusion.

FSH stimulates granulosa cells in ovarian follicles to synthesize aromatase, which converts androgens produced by thecal cells to estradiol. FSH peaks at the same time as the LH surge that causes ovulation. FSH then remains low throughout the luteal phase, preventing the development of new follicles. FSH levels were measured in the serum sample. Estradiol is a steroid hormone associated with the female reproductive organs and is responsible for the development of female sexual characteristics.

- Changes in the levels of cytokines and cellular senescence biomarkers:
  - The concentration of cytokines (TNFa, IFN-γ, IL1, IL-6, IL-8, IL-4, IL-10, IDO) in plasma will be analyzed at the time of intervention and 1 month, 3 months and 6 months after the first AD-MSC infusion using Luminex technology. This will reflect the inflammation status of the body to study the immunoregulatory capacity of AD-MSCs.
  - Signs of aging, including plasma expression of plasminogen activator inhibitor-1 (PAI-1) and increased gene expression of p16 and p21, will be measured at the time of intervention and 6 and 12 months after the first AD-MSC infusion via Luminex technology and real-time PCR, respectively. The molecular analysis might provide a hint for a potential effect of the cell therapy.

***Data collection and management***

All data obtained during the study will be recorded in the patients’ medical reports and the CRF, which will be checked frequently by a quality control officer in the Vinmec Times City International Hospital and Vinmec Scientific Research board for accuracy and consistency. The data in the CRF will be transferred to RedCap software within seven days and cross-checked by the research team. The data from each patient will be collected at six time points during the study, including during the screening period, at baseline, day 30±7, day 90±7, day 180±14 and day 365±14 after the treatment administration (Group A); day 30±7, day 90±7, day 180±14, day 270±14 and day 545±14 (Group B). The data obtained during this clinical trial will be disseminated with permission from the funding body and principal investigator through national and international conferences, peer-reviewed publications, and scientific reports.

***Statistical analysis strategy***

Descriptive statistics included the frequency, percentage, mean, and standard deviation to describe the research subjects' characteristics. Statistical analysis of the FSFI score, quality of life score, FSH levels, and estradiol levels between groups was performed using ANOVA with Tukey’s HSD tests and a two-tailed Wilcoxon rank-sum test with R Program (Version 1.2.5042). A mixed-effect analysis model and paired comparison analysis will be used to analyze variables including FSFI score, quality of life score, FSH levels, and estradiol (E2) levels at assessment time points to compare with before intervention. GraphPad Prism (Version 8.4.3) will be used to create grouped box-and-whisker graphs. The results with p values <0.05 were considered statistically significant.

***Patient and public involvement***

The patients and the public were not involved in our research's design, conduct, reporting, or dissemination plans.

**References**

Bourin, Philippe, Bruce A. Bunnell, Louis Casteilla, Massimo Dominici, Adam J. Katz, Keith L. March, Heinz Redl, J. Peter Rubin, Kotaro Yoshimura, and Jeffrey M. Gimble. 2013. “Stromal Cells from the Adipose Tissue-Derived Stromal Vascular Fraction and Culture Expanded Adipose Tissue-Derived Stromal/Stem Cells: A Joint Statement of the International Federation for Adipose Therapeutics and Science (IFATS) and the International Society for Cellular Therapy (ISCT).” *Cytotherapy* 15 (6): 641–48. https://doi.org/10.1016/j.jcyt.2013.02.006.

Chung, Seok Jong, Tae Yong Lee, Yang Hyun Lee, KyoungWon Baik, Jin Ho Jung, Han Soo Yoo, Chang Jae Shim, et al. 2021. “Phase I Trial of Intra-Arterial Administration of Autologous Bone Marrow-Derived Mesenchymal Stem Cells in Patients with Multiple System Atrophy.” Edited by Stefan Arnhold. *Stem Cells International* 2021 (October): 1–10. https://doi.org/10.1155/2021/9886877.

Dominici, M., K. Le Blanc, I. Mueller, I. Slaper-Cortenbach, F.C Marini, D.S. Krause, R.J. Deans, A. Keating, D.J. Prockop, and E.M. Horwitz. 2006. “Minimal Criteria for Defining Multipotent Mesenchymal Stromal Cells. The International Society for Cellular Therapy Position Statement.” *Cytotherapy* 8 (4): 315–17. https://doi.org/10.1080/14653240600855905.

Harlow, Siobán D., Margery Gass, Janet E. Hall, Roger Lobo, Pauline Maki, Robert W. Rebar, Sherry Sherman, Patrick M. Sluss, Tobie J. de Villiers, and STRAW 10 Collaborative Group. 2012. “Executive Summary of the Stages of Reproductive Aging Workshop + 10: Addressing the Unfinished Agenda of Staging Reproductive Aging.” *Menopause (New York, N.Y.)* 19 (4): 387–95. https://doi.org/10.1097/gme.0b013e31824d8f40.

Hoang, Van T., Quynh-Mai Trinh, Dam Thi Minh Phuong, Hue Thi Hong Bui, Le Minh Hang, Nguyen Thi Hong Ngan, Nguyen Thi Tuyet Anh, et al. 2020. “Standardized Xeno- and Serum-Free Culture Platform Enables Large-Scale Expansion of High-Quality Mesenchymal Stem/Stromal Cells from Perinatal and Adult Tissue Sources.” *Cytotherapy*, October, S1465324920308562. https://doi.org/10.1016/j.jcyt.2020.09.004.

Mendicino, Michael, Alexander M. Bailey, Keith Wonnacott, Raj K. Puri, and Steven R. Bauer. 2014. “MSC-Based Product Characterization for Clinical Trials: An FDA Perspective.” *Cell Stem Cell* 14 (2): 141–45. https://doi.org/10.1016/j.stem.2014.01.013.

Meston, Cindy M. 2003. “Validation of the Female Sexual Function Index (FSFI) in Women with Female Orgasmic Disorder and in Women with Hypoactive Sexual Desire Disorder.” *Journal of Sex & Marital Therapy* 29 (1): 39–46. https://doi.org/10.1080/713847100.

Mohamed, Sara Azhari, Linda Howard, Veronica McInerney, Amjad Hayat, Janusz Krawczyk, Sean Naughton, Andrew Finnerty, et al. 2020. “Autologous Bone Marrow Mesenchymal Stromal Cell Therapy for ‘No-Option’ Critical Limb Ischemia Is Limited by Karyotype Abnormalities.” *Cytotherapy* 22 (6): 313–21. https://doi.org/10.1016/j.jcyt.2020.02.007.

Nguyen Thanh, Liem, Phuong T. M. Dam, Hoang - Phuong Nguyen, Tan - Sinh Thi Nguyen, Huong Minh To, Hung Ba Nguyen, Ngoc - Anh Luu, and Duc M. Hoang. 2021. “Can Autologous Adipose-Derived Mesenchymal Stem Cell Transplantation Improve Sexual Function in People with Sexual Functional Deficiency?” *Stem Cell Reviews and Reports*, June. https://doi.org/10.1007/s12015-021-10196-w.

Randolph, John F., Huiyong Zheng, MaryFran R. Sowers, Carolyn Crandall, Sybil Crawford, Ellen B. Gold, and Marike Vuga. 2011. “Change in Follicle-Stimulating Hormone and Estradiol Across the Menopausal Transition: Effect of Age at the Final Menstrual Period.” *The Journal of Clinical Endocrinology & Metabolism* 96 (3): 746–54. https://doi.org/10.1210/jc.2010-1746.

Rosen, C. Brown, J. Heiman, S. Leib, R. 2000. “The Female Sexual Function Index (FSFI): A Multidimensional Self-Report Instrument for the Assessment of Female Sexual Function.” *Journal of Sex & Marital Therapy* 26 (2): 191–208. https://doi.org/10.1080/009262300278597.

Utian, Wulf H., Jeffrey W. Janata, Sheryl A. Kingsberg, Mark Schluchter, and James C. Hamilton. 2018. “The Utian Quality of Life (UQOL) Scale: Development and Validation of an Instrument to Quantify Quality of Life through and beyond Menopause.” *Menopause* 25 (11): 1224–31. https://doi.org/10.1097/GME.0000000000001223.

Wiegel, Markus, Cindy Meston, and Raymond Rosen. 2005. “The Female Sexual Function Index (FSFI): Cross-Validation and Development of Clinical Cutoff Scores.” *Journal of Sex & Marital Therapy* 31 (1): 1–20. https://doi.org/10.1080/00926230590475206.
